# Sequence-defined phosphoestamers for selective inhibition of the KRAS^G12D^/RAF1 interaction†

**DOI:** 10.1039/d4sc07218a

**Published:** 2024-11-18

**Authors:** Bini Claringbold, Steven Vance, Alexandra R. Paul, James Williamson, Michelle D. Garrett, Christopher J. Serpell

**Affiliations:** a School of Chemistry and Forensic Science, University of Kent Canterbury Kent CT2 7NH UK; b Cancer Research UK Scotland Institute Glasgow G61 1BD UK; c School of Pharmacy, University College London 29-39 Brunswick Square London WC1N 1AX UK chris.serpell@ucl.ac.uk; d School of Biosciences, University of Kent Canterbury Kent CT2 7NJ UK M.D.Garrett@kent.ac.uk

## Abstract

RAS proteins are the most frequently mutated in cancer, yet they have proved extremely difficult to target in drug discovery, largely because interfering with the interaction of RAS with its downstream effectors comes up against the challenge of protein–protein interactions (PPIs). Sequence-defined synthetic oligomers could combine the precision and customisability of synthetic molecules with the size required to address entire PPI surfaces. We have adapted the phosphoramidite chemistry of oligonucleotide synthesis to produce a library of nearly one million non-nucleosidic oligophosphoester sequences (phosphoestamers) composed of units taken from synthetic supramolecular chemistry, and used a fluorescent-activated bead sorting (FABS) process to select those that inhibit the interaction between KRAS^G12D^ (the most prevalent, and undrugged, RAS mutant) and RAF, a downstream effector of RAS that drives cell proliferation. Hits were identified using tandem mass spectrometry, and orthogonal validation showed effective inhibition of KRAS^G12D^ with IC_50_ values as low as 25 nM, and excellent selectivity over the wild type form. These findings have the potential to lead to new drugs that target mutant RAS-driven cancers, and provide proof-of-principle for the phosphoestamer chemical platform against PPIs in general – opening up new possibilities in neurodegenerative disease, viral infection, and many more conditions.

## Abstract 2

Protein–protein interactions (PPIs) drive numerous diseases. We present a new way to inhibit PPIs using high-throughput automated selection of sequence-defined polyphosphoesters, taking the oncogenic mutant KRAS/RAF interaction as a model.

## Graphical abstract

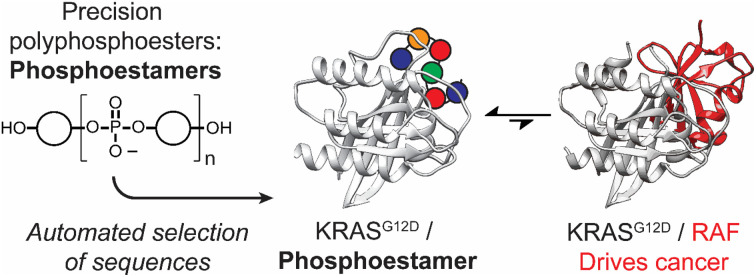

## Introduction

RAS proteins are small GTPases with a GTP-bound “active” state (RAS-GTP) and a GDP-bound “inactive” state (RAS-GDP)^1^ which they cycle between. When in the active conformation, RAS interacts with downstream effector pathways, such as RAF-MEK-ERK, RalGDS and PI3K-AKT-mTOR, to drive proliferative signalling.^2,3^ Kirsten Rat Sarcoma (KRAS) is the most frequently mutated of the RAS family of proteins, accounting for approximately 75% of RAS mutations.^4^ Within KRAS, 98% of the mutations are seen at the G12, G13, or Q61 positions, which lock the protein in the GTP conformation, and hence promote tumourigenesis, but the G12D mutation is the most prevalent overall.^5–8^ KRAS^G12D^ is commonly found in pancreatic,^9^ colorectal,^10^ and lung cancers,^11^ which are associated with poor prognosis in patients^12^ and have high rates of mortality.^13^

The difficulty in drug discovery for RAS is that the only obvious pocket for a small molecule is occupied by GDP or GTP which are both strongly bound,^14^ and present at high cellular concentrations,^15^ making their replacement difficult to envisage. The downstream activity of RAS is driven through protein–protein interactions (PPIs), which involve large surfaces that are relatively flat and featureless compared with the clefts that medicinal chemists classically target.^16^ In the case of RAS, the interaction surfaces lack even well-defined 3D features which could be addressed with compounds such as α-helix mimics.^17^ Nonetheless, small molecule inhibitors have been found which exploit the nucleophilicity of cysteine in the G12C mutant, combined with a less-obvious binding site, which have been approved for the treatment of non-small cell lung cancer. However, this mutant is only present in 12% of such cancers, and resistance has been observed to develop rapidly.^18,19^ While there has been some progress with small molecule G12D inhibitors, such as Mirati Therapeutics MRTX1133,^20^ there is still a great need for more drug discovery research in this area, particularly in the light of drug resistance.

Larger molecules could be used to inhibit PPIs, and indeed there are advances based upon natural sequenced polymers/oligomers, including antibodies,^21^ peptides,^22^ and aptamers,^23^ which have been discovered through selection methodologies. The disadvantages of using biomolecular chemistry are that chemical diversity is fundamentally limited, and that it is recognised by biological processes, which can result in degradation^24^ and/or immune response.^25^ Synthetic foldamers which can display a programmable set of functional groups can circumvent these problems, but are currently best suited as mimics of secondary structures with prior knowledge of which groups should be displayed.^26,27^

Our approach is to create larger synthetic sequence-defined molecules which could cover a significant amount of protein surface area, without bias towards any particular protein substructure. To ensure that uniform macromolecules (as opposed to disperse polymers) can be obtained, we have adapted the automated phosphoramidite chemistry used in oligonucleotide synthesis^28,29^ which is capable of >150 couplings,^30^ but we have employed non-nucleosidic monomers, to obtain phosphoestamers: that is abiotic, uniform oligo- or polyphosphoesters.^31–33^ Lengths of up to 104 monomers have been achieved this way by Lutz, illustrating that stepwise yields can be just as good as those of conventional oligonucleotides.^34^

We herein report the synthesis of a phosphoestamer library, and identification of active sequences through selection by fluorescent activated bead sorting (FABS), which disrupt the interaction between KRAS^G12D^ in its GTP form and the RAS binding domain of C-RAF (RAF1-RBD) with IC_50_ values as low as 25 nM. The stringent process means these phosphoestamers are selective and do not bind either the equivalent wild-type KRAS, nor the GDP-hosting form. These results provide proof-of-principle that phosphoestamers can be effective at blocking medically important protein–protein interactions. While there are potential pharmacokinetic challenges associated with large, polyanionic compounds, these can be overcome, as seen in the field of oligonucleotide therapeutics. Phosphoestamers therefore have the potential to be a transformative technology platform across cancer and other diseases.

## Results

### General overview of the strategy

**Fig. 1:**
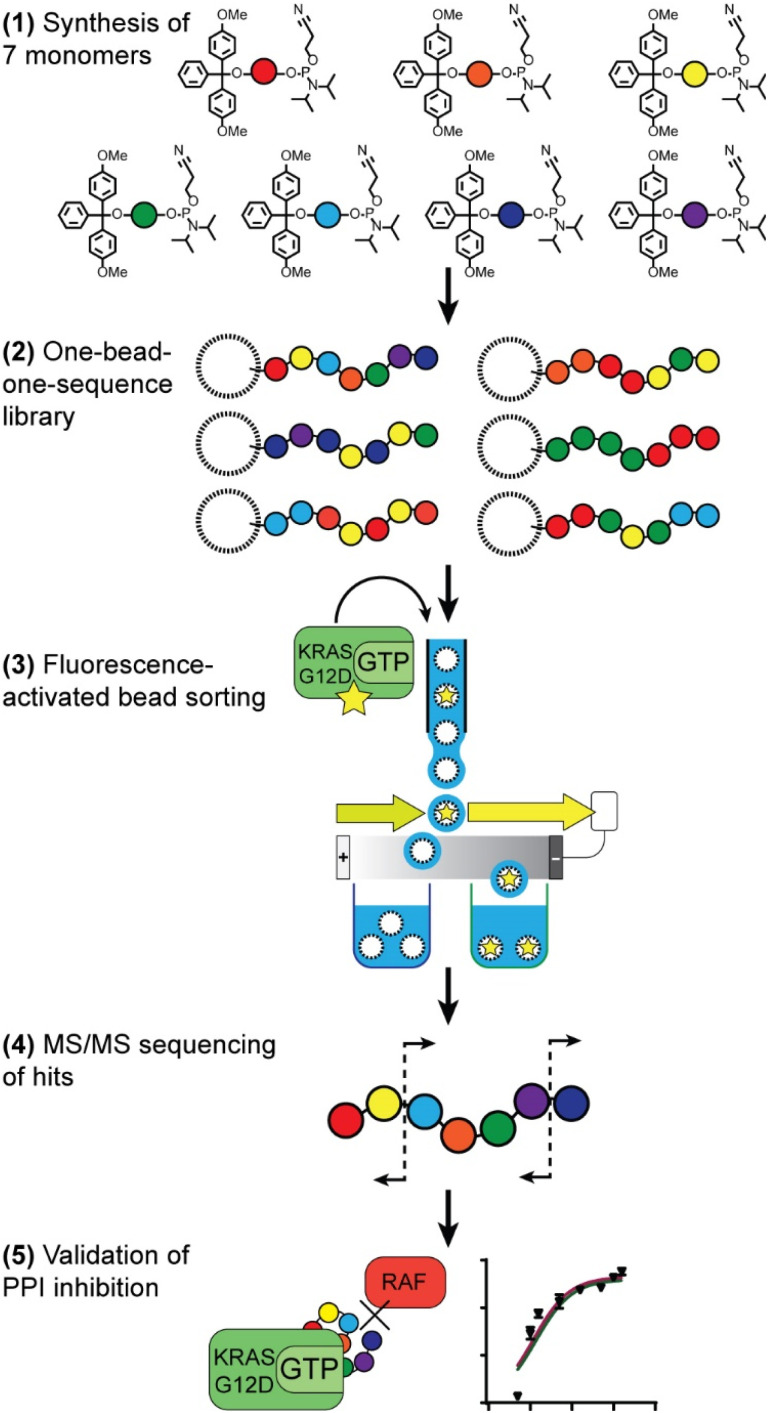
Overview of route to selection of phosphoestamers for PPI inhibition.

Our route to selection of phosphoestamers for PPI inhibition, exemplified here for KRAS^G12D^ in the GTP bound form and RAF (Fig. 1), has five key steps: (1) choice and synthesis of phosphoramidite monomers; (2) synthesis of the one-bead-one-sequence library; (3) rounds of fluorescence-activated bead sorting (FABS) for the selection of phosphoestamers that disrupt PPIs; (4) sequencing of selected phosphoestamers by LC-MS/MS; and (5) resynthesis and validation of these molecules in an orthogonal assay.

### Monomer and library synthesis

**Fig. 2:**
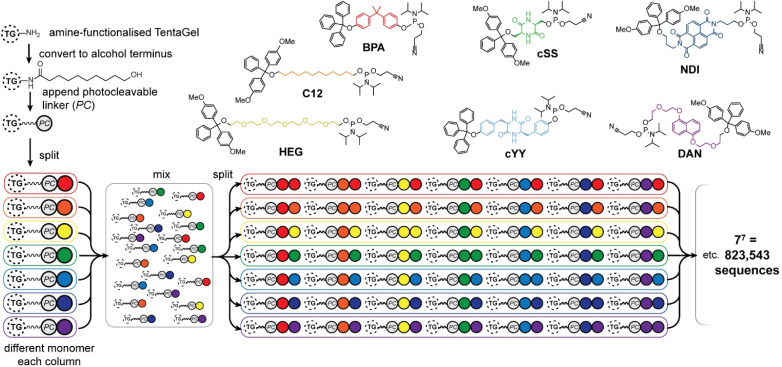
Phosphoramidites used for the phosphoestamer library, and preparation of the library on TentaGel® (TG) beads with a photocleavable (PC) linker by split-and-mix synthesis.

Seven phosphoramidite monomers (Fig. 2) were selected for use in the phosphoestamer library. The synthesis requires monomers to be based upon diols which are then protected at one hydroxyl with a dimethoxytrityl (or trityl if phenolic) group, followed by activation at the second using 2-cyanoethyl *N*,*N*-diisopropylchlorophosphoramidite (full procedures and data, ESI Section 2.2†). This yields monomers which can be linked using standard automated oligonucleotide synthesis chemistry. The monomers were chosen such that they cover a wide range of potential supramolecular interactions. BPA (based upon the diol bisphenol A) and C12 (dodecanediol) provide hydrophobic regions within the phosphoestamer, with BPA being rigid while C12 is flexible. HEG (hexaethylene glycol) is hydrophilic. Patterns of C12 and HEG have been shown to direct supramolecular chemistry in phosphoestamers.^32,35,36^cSS (cyclic di-serine) and cYY (cyclic di-tyrosine) are diketopiperazines based upon amino acids which form a rigid structure and are able to act as both hydrogen bond donors and acceptors.^37,38^NDI (naphthalene diimide) and DAN (dialkoxynaphthalene) are capable of π–π interactions, and in particular form a donor (DAN)/acceptor (NDI) pair which also enables folding,^39^ including in phosphoestamers.^33,40^ All phosphoramidite monomers were successfully synthesised, except C12 and HEG which were available commercially.

The one-bead-one-sequence phosphoestamer library was constructed using split-and-mix techniques^41^ (Fig. 2, ESI Sections 2.3 and 2.4†). Creating 7mers of all the combinations of the monomers produced 7^7^ = 823 543 unique full-length sequences, plus any sequences that did not go to completion. This library size was chosen based upon conservative estimates of synthetic and analytical capacity from a previous project.^42^ Synthesis of the library was completed using automated phosphoramidite synthesis on TentaGel® M. NH_2_ monosized (10 μm) Amino TentaGel Microspheres (TG-beads); TG-beads have a polystyrene backbone with a PEG spacer and are chemically inert, making them suitable to phosphoramidite addition.^43^ The TG-beads were modified with 10-hydroxydecanoic acid to create hydroxy TG-beads and were swelled in dichloromethane before a photocleavable linker was attached to allow for UV-activated liberation of sequences from the TG-beads after fluorescent selection.^44^ The beads were then split for the first round of monomer addition. After each monomer was added to an individual pool, the library was mixed and split out again for the second monomer addition, creating 49 different combinations in the second step, before being mixed again as the cycle continues. The resultant phosphoestamer library contained over 200 million individual TG-beads, giving on average 268 beads for each of the 7^7^ sequences, each displaying 10^11^ copies of that specific phosphoestamer sequence (Fig. 2). The trityl monitor was used to monitor the efficiency of each coupling, with near-quantitative results at every step, consistent with previous reports.^32,34^

### Selection by fluorescence-activated bead sorting

Fluorescent-activated bead sorting (FABS) is a methodology that allows for the selection of the highest binding phosphoestamer to a specified protein target using a flow cytometer. Flow cytometry has previously been used for the selection and optimisation of aptamers^42,45,46^ and inhibitors of small GTPases such as Rho and Rab.^47^ We used several selection steps to identify phosphoestamers that bind to KRAS^G12D^-GMPPnP (non-hydrolysable analogue of GTP) and disrupt any interaction between KRAS^G12D^-GMPPnP and RAF1-Ras binding domain (RAF1-RBD). Proteins were produced (ESI Section 3†) and fluorescent labels were attached meaning that the binding affinity between bead-confined phosphoestamers and proteins is correlated to the fluorescence displayed by the bead in FABS analysis. Gating can then be used to separate beads above or below any chosen fluorescence intensity, indicating higher or lower binding by the sequence on that bead. The proteins used in the FABS selection were expressed with a biotin tag, which was then used as a linker to fluorophore-labelled streptavidin (STV). KRAS^G12D^-GMPPnP and KRAS^G12D^-GDP were tagged with fluorescein-STV, and RAF1-RBD with rhodamine Red™-X-STV.

**Fig. 3:**
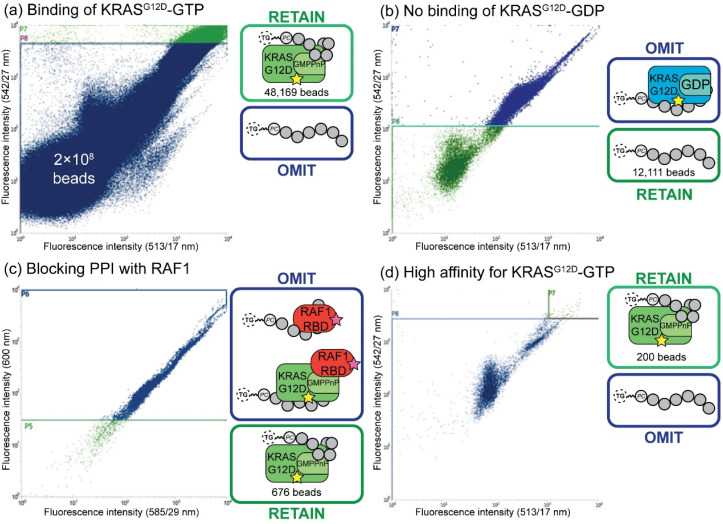
FABS selection of top binding phosphoestamers. Yellow star = fluorescein label; pink star = rhodamine label. (a) Initial selection step, selecting for phosphoestamers that bind to KRAS^G12D^-GMPPnP. (b) Second selection step, selecting for phosphoestamers that do not bind to KRAS^G12D^-GDP. (c) Third selection step, selecting for phosphoestamers that do not bind to RAF1-RBD. (d) Fourth selection step, selecting top phosphoestamers that bind to KRAS^G12D^-GMPPnP.

Selection of the phosphoestamer library for KRAS^G12D^-GTP/RAF1-RBD PPI inhibition employed four rounds of FABS selection (Fig. 3, full data and analysis, ESI Section 4†). In Round 1 the phosphoestamer library was incubated with enough fluorescein-tagged KRAS^G12D^-GMPPnP to cover 4% of the library; only phosphoestamers with a high affinity for KRAS^G12D^-GMPPnP would therefore acquire detectable fluorescence, and thus be retained for round 2 (Fig. 3a), giving 48 169 beads of the original 2 × 10^8^. KRAS^G12D^-GMPPnP was removed from the selected beads by washing in preparation for the next round. The pool was then incubated with fluorescein-tagged KRAS^G12D^-GDP. Beads which display a strong fluorescent signal would be bound to KRAS^G12D^-GDP, and were therefore removed in this round of FABS (Fig. 3b), leaving 12 111 library beads: since KRAS-GDP does not interact with RAF, it is not directly oncogenic. KRAS^G12D^-GDP was removed by washing, and the remaining library was incubated with fluorescein-tagged KRAS^G12D^-GMPPnP and a 3-fold excess of RAF1-RBD (rhodamine Red™-X tagged). Here, any phosphoestamers from the library that had a high fluorescent signal using the 585/29 and 600 nm bandpass filters (indicating rhodamine) either had a high affinity for RAF1-RBD itself or, more likely given prior selection rounds, bound KRAS^G12D^-GTP in a manner which did not prevent the GTPase from also binding RAF1-RBD. Conversely, those beads which did not acquire rhodamine fluorescence must inhibit the PPI since their binding of KRAS was selected for in the first round (Fig. 3c); this was validated through checking fluorescein fluorescence, which gave high readings (Fig. S44, ESI†). The result of this third selection was 676 library beads and so a fourth selection round was used to identify only those with highest affinity for KRAS^G12D^-GMPPnP. The selected beads were washed again to remove any remaining proteins and incubated with enough fluorescein-tagged KRAS^G12D^-GMPPnP to cover 50% of the remaining library. For the final selection, 200 beads of the highest fluorescence were sorted such that each individual bead was placed in an individual well of a 96-well plate (Fig. 3d). The phosphoestamers were then cleaved from the TG-beads *via* the photocleavable linker.

### Sequencing of hit phosphoestamers by mass spectrometry

Phosphoestamers were sequenced and identified with a Q-TOF nanospray LC-MS/MS method. A commercially purchased 7-base DNA oligomer was used to identify the limit of detection and observe patterns in how phosphoestamers of this length could fragment. These results showed the phosphoestamers were most likely to be detected as [M − 2H]^2−^ parent ions, and MS/MS identified c- and y-ions^48^ as the most predominant. Of the 200 top phosphoestamers selected from FABS, 21 selected at random (according to instrumental capacity) were prepared for LC-MS/MS analysis, and 6 phosphoestamers (O1–O6) produced data which could be fully interpreted (Table 1, full data analysis ESI Section 5†). MS/MS data revealed molecular ions which fell within the expected phosphoestamer library range (1669.08–3125.43 Da), with the exception of O6 (*M* = 1402.24) which represents a truncation. Data from O1 showed not only the common [M − 2H]^2−^ parent ion, but also a smaller [M − H]^−^ ion at 1710.693 *m*/*z*; this provided two separate sets of MS/MS data that could be analysed and compared when identifying the sequence, assisting in validation of the workflow. Sequencing was performed using RoboOligo, a programme designed for the analysis of tandem mass spectrometry data of oligonucleotides.^49^

**Table 1:** Phosphoestamer masses detected *via* LC-MS/MS

Phosphoestamer	*m*/*z* detected	Neutral molecular mass (Da)
O1	854.978 [M − 2H]^2−^	1711.970
	1710.963 [M − H]^−^	
O2	992.314 [M − 2H]^2−^	1986.642
O3	911.342 [M − 2H]^2−^	1824.698
O4	971.321 [M − 2H]^2−^	1944.656
O5	971.288 [M − 2H]^2−^	1944.590
O6	700.120 [M − 2H]^2−^	1402.240

**Fig. 4:**
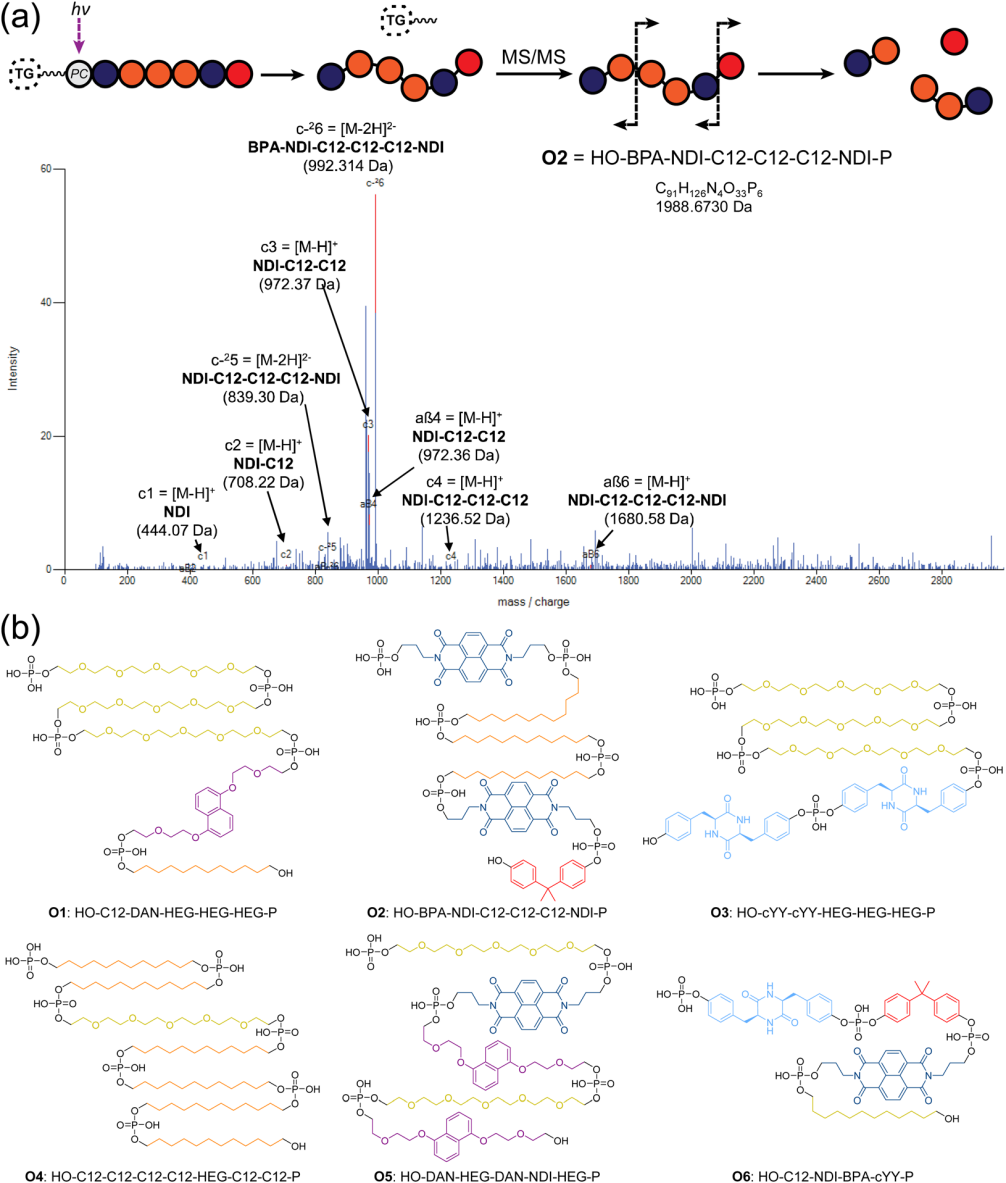
(a) RoboOligo analysis of O2. Other major peaks are indexed on alternative fragments (see ESI Section 5.2†). (b) Sequences of top binding phosphoestamers. Sequences are given by analogy with nucleic acid conventions (5′ to 3′) meaning that the first monomer listed is the last one added during chemical synthesis. This is evident from the location of the terminal phosphate which exists in the MS/MS spectra as a result of the photocleavage reaction, leading to the HO- and -P (phosphate) termini.

Fig. 4a shows the RoboOligo analysis of O2, which was identified as **NDI-C12-C12-C12-NDI-BPA**. Examining all the sequences selected (Fig. 4b), there were some common patterns identified, such as the multiple adjacent monomers of both C12 in O2 and O4, and of HEG in O1 and O3. Every initial monomer used was found in at least one phosphoestamer sequence, except cSS which was not seen in any top binder analysed. Of the sequences identified only one (O4) was a full-length 7-mer, with O1–O5 being 5/6mer oligomers and O6 being a tetramer. Given that monitoring trityl groups during synthesis showed that the couplings were successful to the end, and having used a redundancy of 268, at least one instance of each full-length sequence should be present. It is therefore likely that these smaller phosphoestamers are better binders compared to the 7mers. We have observed the selection of optimal sequences arising from synthetic inefficiencies previously,^42^ and suspect that the incidence of beads displaying an entire population of truncated sequences could occur through imperfect distribution in the flow of reagents over the beads.

### Validation of PPI inhibition by phosphoestamers

**Fig. 5:**
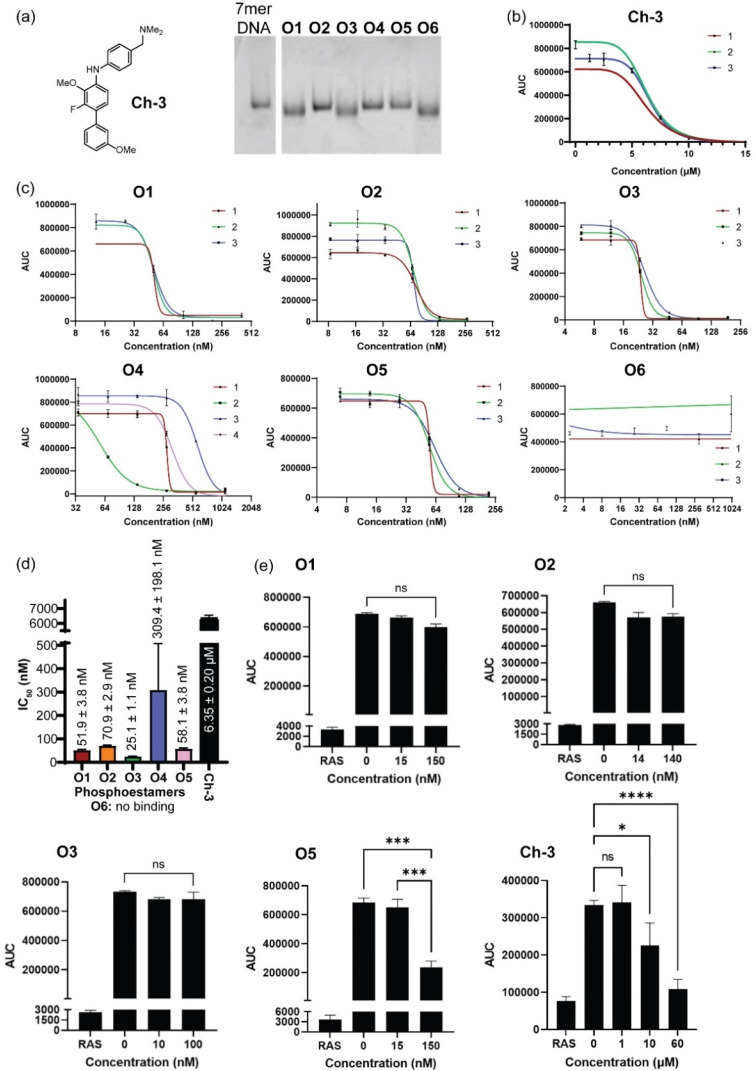
(a) Positive control compound Ch-3, and pure phosphoestamers O1–O6 characterised by polyacrylamide gel electrophoresis. (b) KRAS^G12D^/RAF1-RBD interaction assay results for Ch-3. Data collected using area under the curve (AUC) of GFP emission spectra between 490 and 540 nm. Numbers 1–3 indicate biological repeats. (c) KRAS^G12D^/RAF1-RBD interaction assays results for O1–O6. (d) IC_50_ values for Ch-3 and O1–O6 calculated from assays. (e) Effect upon KRAS^WT^/RAF1-RBD interaction of Ch-3 and top scoring phosphoestamers screened at concentrations up to 3 × IC_50_ for KRAS^G12D^/RAF1-RBD.

Phosphoestamers O1–O6 were resynthesised on a 1 μmol scale using the DNA synthesiser and the yields were determined by manually cleaving the final DMT protecting group of each molecule and quantifying the DMT cation by UV-visible spectroscopy (ESI Section 2.4†). The achievement of desired length and purity was confirmed by polyacrylamide gel electrophoresis (Fig. 5a). The resynthesised phosphoestamers were purified away from smaller molecules using C18 spin tips. To ensure the assay was viable, a positive control Ch-3 (Fig. 5a) known to disrupt KRAS^G12D^ interactions^50^ was synthesised. An assay was developed (ESI Section 6†) in which polystyrene 96-well plates were coated by overnight incubation with KRAS^G12D^-GMPPnP.^51^ The wells were then washed with a blocking solution before incubation with phosphoestamers and RAF1-RBD-GFP. Any KRAS sites not blocked by the phosphoestamers would interact with the RAF1-GFP and result in a fluorescent signal which would be detected. In this assay, we first established that both KRAS^G12D^-GMPPnP and RAF1-RBD-GFP were required to give a fluorescence signal. The assay was then conducted at varying concentrations of Ch-3, giving a resultant IC_50_ of 6.35 ± 0.20 μM (Fig. 5b), consistent with reported assays performed with the same compound.^50^

Conducting the same assays with the phosphoestamers (Fig. 5c and d) showed that O1, O2, O3 and O5 had IC_50_ values below 100 nM: 20–250 times smaller than the positive control 8. O6 showed no change in fluorescent signal across three repeats, and so is unlikely to have any effect on the PPIs between KRAS^G12D^ and RAF. O6 is the smallest phosphoestamer, and it is possible that the MS/MS has only detected a fragment of a whole chain that does not successfully disrupt the PPIs on its own, or that some multivalency effect which was in operation on the beads but cannot work with isolated strands.

O3 had the lowest IC_50_ value, at 25.14 ± 1.06 nM; suggesting the strongest affinity for KRAS^G12D^-GMPPnP. O1 had a similar structural motif to O3 and had the second lowest IC_50_ at 51.94 ± 3.75 nM. The HEG-HEG-HEG sequence could be key to improving binding to KRAS^G12D^-GMPPnP compared to the other oligomers. O5 had a similar IC_50_ to O1, 58.08 ± 3.78 nM, but the only similarity between these two is a HEG-DAN subsequence within the phosphoestamers. O2 was the only 6-mer and had a higher IC_50_, 70.87 ± 2.88 nM, which could suggest that 5-mer phosphoestamers do have a greater affinity for KRAS proteins compared to the longer chains. The only full length (7-mer) phosphoestamer was O4, and like O2 had a much larger IC_50_, 309.38 ± 198.09 nM; the standard deviation was large meaning this molecule potentially does not bind or disrupt interactions consistently – this is unsurprising given its very flexible nature, which may only bind when multivalency is provided on a bead surface.

Since four phosphoestamers were determined to have consistent dissociation activity between KRAS^G12D^-GMPPnP and RAF1-GFP, we then used the same assay to determine whether these molecules would have any activity against KRAS^WT^-GMPPnP or whether they would be selective for the mutant form (Fig. 5e). Testing ‘high’ and ‘low’ concentrations of O1, O2, O3, and O5 (approximately IC_50_ ÷ 3 and IC_50_ × 3 respectively), it was found that only O5 caused a decrease in fluorescent signal, indicating disruption of the KRAS^WT^-GMPPnP/RAF1-GFP interaction, and weaker selectivity. An IC_50_ for the WT PPI was determined for O5 at 125.61 ± 8.45 nM, more than twice that of the value for KRAS^G12D^-GMPPnP, meaning that even O5 has some selectivity for KRAS^G12D^-GMPPnP over the WT.

Overall, the FABS selection process was successful in providing potential inhibitors of the KRAS^G12D^/RAF1 PPI, with these phosphoestamers having a much stronger binding affinity for KRAS^G12D^ compared to the positive control used. Additionally, three of these phosphoestamers are selective for the mutant active form KRAS^G12D^ over KRAS^WT^.

## Discussion

We have combined several different techniques – phosphoramidite synthesis, one-bead-one-compound library synthesis, fluorescence-activated bead sorting, and tandem mass spectrometry – to create a unique methodology for the selection of novel phosphoestamers that selectively inhibit protein–protein interactions between mutant KRAS^G12D^ and RAF1. FABS was first investigated some time ago,^52,53^ but has been largely neglected until recently because of problems such as autofluorescence^54^ and insufficient loading of the beads. More recently, using modern instruments, these problems are minimised,^55,56^ while FABS provides unique features such as simultaneous analytical readout of affinity, customisable selection gates, and multi-parametric selection.^57,58^ The potential of the method is further supported here, and with our previous work on identifying modified nucleic acid aptamers.^42^

The top three final targets (O1, O2 and O3) were inactive against KRAS^WT^ but inhibited KRAS^G12D^ with IC_50_ values of between 25 and 58 nM. Mutations in RAS cause overactive cell signalling, driving 30% of cancers including ∼95% of pancreatic, 45% of colorectal cancers and 32% of lung adenocarcinomas,^59^ and it stands as an extremely important drug target in cancer therapy.^60^ Current examples of KRAS^G12D^-GTP inhibitors work at between 180 nM and 6 μM (ref. 50, 61 and 62) in biochemical assays, and our methodology has exceeded the activity of those compounds. MRTX1133, a highly optimised G12D drug in clinical trials has an IC_50_ of 5 nM, but binds to the inactive GDP-form of the protein.^20^ We have not undertaken any chemical optimisation of phosphoestamers, but nonetheless have obtained strong inhibitors. Our mechanistic aim differs in that our selection was set up not for binding to a particular site, but for blocking of a specific PPI – in this case one which only the GTP form participates in. This is important because it means that in principle, our method could be used to generate phosphoestamers addressing any other PPI of interest, thus opening up access to modulating mechanisms in diseases as diverse as neurodegeneration^63^ and viral infection.^64^

Looking forward to potential applications which would ideally be in medicine, it will be immediately clear to any medicinal chemist that the molecules selected are not classically ‘drug-like’ in their size, polarity, or rigidity: phosphoestamers are large, polyanionic, and very flexible, which means that their ability to cross membranes is probably minimal, but this does not mean that they should be dismissed for drug discovery. These molecules are physicochemically related to oligonucleotides, which are now a successful class of drug, operating inside the cell, with the overwhelming majority relying on effects of chemical modification (such as phosphorothioation^65^) rather than on delivery vehicles to achieve this.^66^ Nonetheless, vehicles such as lipid nanoparticles exist,^67^ and we are in the process of exploring these possibilities.

In comparison with existing technologies for inhibition of PPIs, phosphoestamers have a number of advantages. Use of small molecules requires identification ‘hot spots’ (smaller areas which contribute decisively to the binding energy), the discovery of which is laborious^68^ without guarantee of success.^16^ Nonetheless, the precise design of small molecule structure means that they can be exquisitely optimised when hits are found. The phosphoestamer selection platform does not require hot spots, but is just as amenable to precise and arbitrary structural modification. Current alternatives to small molecules are biopharmaceuticals (peptides, antibodies, and nucleic acid aptamers) or derivatives thereof. A general drawback of using such systems is that their chemistry is the same as that used by the body, meaning that they can be recognised by immune processes, or be subject to enzymatic degradation. The extent varies: peptides^69^ are attractive for their ease of chemical modification^70^ which permits fine-tuning of target engagement and pharmacokinetics, but are recognised by proteases^71^ and the immune system;^72^ antibodies^73^ have good biostability^74^ and only residual immunogenicity in humanised versions,^75^ but are only minimally chemically customisable;^76^ while aptamers^77^ can be modified in a range of ways^78^ and are negligibly immunogenic,^79^ but rapid degradation and elimination is a problem.^80^ For phosphoestamers, there are physiological nucleases which might pose a degradation risk, but even for nucleic acids these can be circumnavigated through modifications,^81^ or indeed through addition of non-natural monomers, including some we have used here.^82^ Toll-like receptors recognize nucleic acids as part of the innate immune system, but these are specific to certain sequences and structures,^83^ and would not be expected to be activated by any oligophosphoester. We can therefore expect that phosphoestamers will be less susceptible to degradation or immune response due their bioorthogonality, but these hypotheses are currently under investigation in our lab.

Phosphoestamers are an interesting class of materials in themselves, displaying sequence-, concentration-, and cation-dependent supramolecular chemistry.^31^ This responsive behaviour, particularly self-assembly could impact therapeutic applications, but in the case of the short oligomers here we have no observed any evidence of that, and due to their potency we are working well below the critical aggregation concentration of much more hydrophobic systems.^32^

In summary, there is a wide scope of opportunities and challenges for ahead phosphoestamers in applied biomedical science.

## Conclusions

We have synthesised and screened a library of phosphoestamers for inhibition of the undrugged mutant KRAS^G12D^-GTP/RAF interaction, through fluorescence-activated bead sorting, identifying six novel molecules through tandem mass spectrometry analysis. Validation assays showed that three of these phosphoestamers show both a high affinity to KRAS^G12D^ that disrupts the interaction with RAF1, and does not affect the equivalent PPI in the wild type protein. This affinity is an improvement upon previously synthesised inhibitors, and if intracellular access can be engineered, it provides leads for development of new types of drugs. It also provides proof-of-concept that this technology platform could be used to identify inhibitors against other difficult protein–protein interactions in cancer and also other disease areas, both inside and outside the cell.

## Data availability

The ESI† contains full experimental details, and additional data for chemical synthesis, protein expression, FABS, mass spectrometry, and assays. The authors have cited additional references within the ESI.†,^84,85^

## Author contributions

B. C. performed the bulk of the experimental investigation (synthetic, selection, analytical) and wrote the first draft of the paper. S. V. provided protein samples and RAS-related expertise. A. R. P. contributed to methodology development. J. W. contributed to molecular characterisation. M. D. G. and C. J. S. conceived, and supervised the project. All authors contributed to manuscript finalisation.

## Conflicts of interest

There are no conflicts to declare.

## Supplementary Material

SC-OLF-D4SC07218A-s001
